# Hypertension and cardiomyopathy associated with chronic kidney disease: epidemiology, pathogenesis and treatment considerations

**DOI:** 10.1038/s41371-022-00751-4

**Published:** 2022-09-22

**Authors:** Jonathan P. Law, Luke Pickup, Davor Pavlovic, Jonathan N. Townend, Charles J. Ferro

**Affiliations:** 1 Institute of Cardiovascular Sciences, University of Birmingham, Birmingham, UK; 2 Department of Renal Medicine, Queen Elizabeth Hospital, University Hospitals Birmingham NHS Foundation Trust, Birmingham, UK; 3 Department of Cardiology, Queen Elizabeth Hospital, University Hospitals Birmingham NHS Foundation Trust, Birmingham, UK

**Keywords:** Prognosis, Cardiac hypertrophy, Chronic kidney disease, Hypertension

## Abstract

Chronic kidney disease (CKD) is a complex condition with a prevalence of 10–15% worldwide. An inverse-graded relationship exists between cardiovascular events and mortality with kidney function which is independent of age, sex, and other risk factors. The proportion of deaths due to heart failure and sudden cardiac death increase with progression of chronic kidney disease with relatively fewer deaths from atheromatous, vasculo-occlusive processes. This phenomenon can largely be explained by the increased prevalence of CKD-associated cardiomyopathy with worsening kidney function. The key features of CKD-associated cardiomyopathy are increased left ventricular mass and left ventricular hypertrophy, diastolic and systolic left ventricular dysfunction, and profound cardiac fibrosis on histology. While these features have predominantly been described in patients with advanced kidney disease on dialysis treatment, patients with only mild to moderate renal impairment already exhibit structural and functional changes consistent with CKD-associated cardiomyopathy. In this review we discuss the key drivers of CKD-associated cardiomyopathy and the key role of hypertension in its pathogenesis. We also evaluate existing, as well as developing therapies in the treatment of CKD-associated cardiomyopathy.

## Introduction

Chronic kidney disease (CKD) and kidney failure with replacement therapy (KFRT) are complex chronic conditions with a combined prevalence of 10–15% worldwide [1–5]. Hypertension is an equally significant global problem and remains one of the most important preventable causes of mortality worldwide [6, 7], with the prevalence expected to rise to 1.56 billion by 2025 [6]. Affecting 67–92% of patients with CKD, hypertension is also the most common comorbidity with increasing prevalence and severity as kidney function progresses [8]. Its pathogenesis and that of CKD are tightly intertwined, with hypertension being both a complication of and driver of kidney disease progression [9–11].

An inverse graded relationship exists between cardiovascular events and mortality with estimated glomerular filtration rate (eGFR), which is independent of age, sex, and other risk factors [12–18]. The proportion of deaths due to heart failure and sudden cardiac death (SCD) increase with progression of CKD, with relatively fewer deaths from atheromatous processes [19–23]. This is also evidenced by trials which show benefit of lipid-lowering therapies in early CKD [24–26], but appear to be ineffective in patients with KFRT [26–28]. This is thought to be the result of the development of CKD-associated cardiomyopathy.

In this state-of-the-art review, we will discuss some of the key drivers of CKD-associated cardiomyopathy and the key role of hypertension in its pathogenesis, and evaluate existing, as well as developing therapies in the treatment of CKD-associated cardiomyopathy.

## CKD-associated cardiomyopathy

**Fig. 1:**
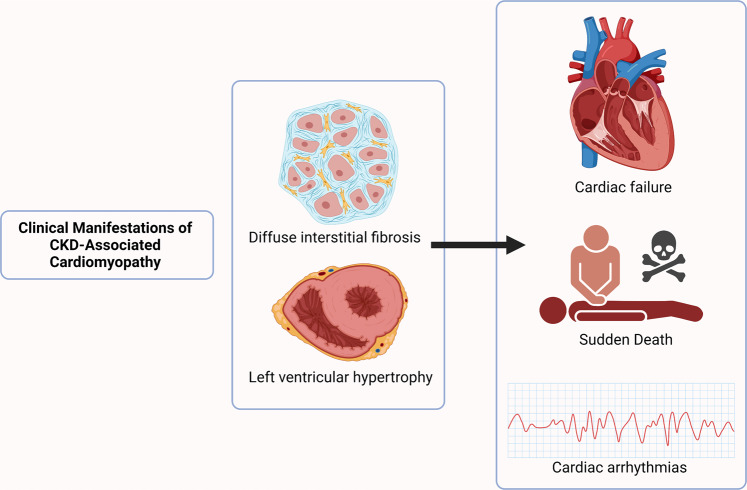
CKD-associated cardiomyopathy. CKD-associated cardiomyopathy is characterised by structural remodelling of the heart. Diffuse interstitial fibrosis and cardiac hypertrophy give rise to cardiac electromechanical dysfunction and increased risk of sudden death.

The concept of CKD-associated cardiomyopathy (Fig. 1) first appeared in the 1980s following reports of common abnormalities in cardiac structure and function in patients with CKD and KFRT [29, 30]. The key features described were:

- Increased left ventricular (LV) mass and left ventricular hypertrophy (LVH),
- Diastolic and systolic LV dysfunction,
- Profound myocardial fibrosis on histology [15, 31–44].

Whilst these features have been predominantly identified in patients with KFRT, who are undoubtedly uraemic, those with mild to moderately reduced eGFR already exhibit structural and functional changes consistent with CKD-associated cardiomyopathy [35]. This is consistent with observational studies reporting a 20% higher risk of death and cardiovascular events in patients with eGFR between 45 and 59 ml/min/1.73 m^2^ compared to those with eGFR greater than 60 ml/min/1.73 m^2^ [14].

### Increased left ventricular mass/left ventricular hypertrophy

**Fig. 2:**
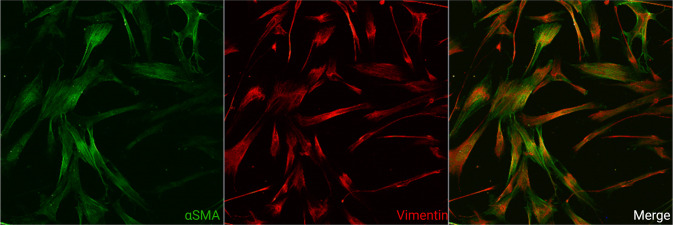
Activated human induced pluripotent stem cell-derived cardiac fibroblasts. Laser scanning confocal image of human induced pluripotent stem cell-derived cardiac fibroblasts activated by transforming growth factor-β stained with antibodies against α-smooth muscle actin (green) and vimentin (red). Every third to fourth cell in the left ventricle is a fibroblast. Most cardiac research has focused on the cardiomyocyte with research on cardiac fibroblasts proving more challenging. (Image provided by Ms Caitlin Hall, Institute of Cardiovascular Sciences, University of Birmingham).

The LV is a main target for end-organ damage in hypertension, resulting from a combination of cardiomyocyte hypertrophy and expansion of the extracellular space caused by interstitial myocardial fibrosis from activated fibroblasts (myofibroblasts; Fig. 2) [15]. In the general population, the prevalence of LVH is between 15 and 21% [45]. The prevalence is significantly greater in patients with hypertension. In a meta-analysis encompassing 37,700 hypertensive patients, LVH was detected by echocardiography in 36–41%, increasing to 58–77% in high-risk patients who had severe or refractory hypertension, type 2 diabetes mellitus, or a history of previous cardiovascular events [46]. However, it should be noted that the relationship between LV mass and blood pressure is continuous, with no true dichotomy, and that any definition of LVH is a useful, but arbitrary way of defining hypertensive target organ damage [47, 48].

LVH is an independent risk factor for cardiovascular morbidity and mortality [49]. Prospective data from large observational studies [45, 50] have demonstrated independent associations between echocardiogram/electrocardiogram-LVH, and an increased risk of cardiovascular events and all-cause mortality. In a large retrospective study of 35,602 patients referred for echocardiography, the presence of LVH increased the risk of all-cause mortality by 2-fold over a mean follow-up of 3.2 years [51]. In the Heart and Soul Study, a cohort of 1016 patients with stable coronary artery disease followed up for 3.5 years, evidence of echocardiographic LVH was associated with a higher overall mortality (25% v 11%) and SCD (6.7% v 2.2%) [52]. Analysed as a continuous variable, every 20-unit increase in LV mass increased the adjusted hazard of overall mortality by 22% and of SCD by 40% [52].

Ambulatory blood pressure measurements more closely correlate with LV geometry abnormalities compared to office blood pressure measurements [53, 54]. Patients with a non-dipping or reverse dipping pattern on 24-h ambulatory monitoring have a higher LV mass and increased prevalence of LVH [55, 56] as well as worse CV outcomes [57, 58]. These abnormal blood pressure circadian rhythms are also more prevalent in patients with CKD and KRFT and are also associated with worse CV outcomes [59].

### Increased left ventricular mass/left ventricular hypertrophy in chronic kidney disease

The connection between CKD, hypertension and LVH was first described by several 19^th^ century physicians. Pioneering British physician Richard Bright (1789–1858) depicted with meticulous detail first observations of LVH present on autopsies of patients with albuminuria [60]. Years later, his successor Sir Samuel Wilks (1824–1911) described pathological changes in the heart and arteries accompanying Bright’s disease [61]. Further publications in the 1850s by Ludwig Traube (1818–56) and William Kirkes (1822–64) introduced the concept of arteriosclerosis and high intra-arterial pressure as a driver of LVH in renal disease, laying the foundation for our understanding of CKD-associated cardiomyopathy [62, 63].

**Fig. 3:**
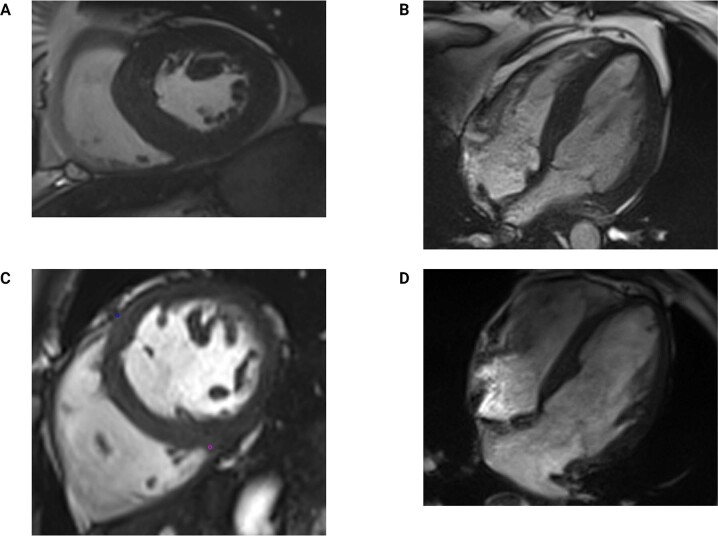
Cardiac magnetic resonance images demonstrating left ventricular hypertrophy and normal dimensions in dialysis patients. Short axis stack image (**A**) and horizontal long axis image (**B**) of a patient on haemodialysis with concentric left ventricular hypertrophy on cardiac magnetic resonance imaging (maximum wall thickness, 15 mm; left ventricular mass 93 g/m^2^). Short axis stack image (**C**) and horizontal long axis image (**D**) of a patient on peritoneal dialysis with normal left ventricular dimensions on cardiac magnetic resonance imaging (maximum wall thickness, 9 mm; left ventricular mass 63 g/m^2^).

Recent imaging studies confirm that increased LV mass and LVH are common manifestations of CKD-associated cardiomyopathy (Fig. 3). LVH is found in 48–84% of patients with pre-dialysis CKD [64–66], and in up to 90% of patients with KFRT receiving haemodialysis [5, 38, 67]. Similarly, LV mass is a continuous variable with a graded association with adverse cardiovascular outcomes and mortality [45, 68–72]. In an analysis of 1,249 patients with predialysis CKD, LVH was associated with a mortality risk of 25 deaths per 1000 person-years [68]. In patients who have LVH at dialysis initiation, the risk of mortality was 1.75-fold greater than those without LVH at 6 years follow-up [5]. Conversely, LVH regression may improve outcomes in patients with KFRT [67].

LVH is associated with an increased risk of arrhythmia, which may explain, in part, the increased incidence of cardiovascular death in CKD [73]. A meta-analysis of 10 studies (27,141 patients) found that patients had 3.4-fold and 2.8-fold greater odds of developing sustained supraventricular tachycardia and ventricular arrhythmias, respectively, in the presence of LVH [74]. Other studies reported a 20% increase in the risk of atrial fibrillation (AF) for every standard deviation increase in LV mass [70]. The presence of LVH in hypertensive patients was a predictor for progression from paroxysmal to permanent AF [71], whilst blood pressure reductions of 7.3/1.8 mmHg resulted in regression of LV mass index with a corresponding 7-fold decline in the prevalence of paroxysmal AF after a mean follow-up of 24 months [75].

### Diastolic and systolic dysfunction

Diastolic dysfunction is highly prevalent in patients with CKD [76] with studies reporting an incidence of 71% in CKD stages 2–4 [64] and up to 85–90% in KFRT [77–79]. Excess myocardial deposition of collagen affects cardiac muscle viscoelasticity, leading to increased ventricular stiffness, impaired myocardial relaxation and diastolic recoil [80]. Diastolic dysfunction is strongly association with increased LV mass and LVH [19], as well as myocardial fibrosis [41, 81], and is associated with increased mortality [78, 81]. Indeed, the American Society of Echocardiography and the European Association of Cardiovascular Imaging considers LVH as an indicator of diastolic dysfunction [82]. Furthermore, the presence of diastolic dysfunction is considered to be a major cause for the frequent presentation of HD patients with pulmonary oedema or intradialytic hypotension with only minor changes in fluid status [19, 81]. Overt LV systolic dysfunction, as manifest by a reduced left ventricular ejection fraction, is relatively uncommon in pre-dialysis CKD, with a reported prevalence of 8% and no association with eGFR [36, 64]. However, several studies have shown changes in LV deformation in early stages of CKD indicating the presence of sub-normal LV systolic function [83–85]. In KFRT, LV systolic dysfunction is very common with a reported prevalence 10–30 times greater than in the general population [86–89].

The presence of LVH has also been associated with the risk of development of systolic dysfunction in hypertensive subjects [90–92]. The 2013 American College of Cardiology Foundation/American Heart Association Guideline for the Management of Heart Failure recognised hypertension and LVH as stage A and B heart failure. The guideline emphasised the progressive nature of heart failure, and the importance of long-term treatment of elevated blood pressure to prevent symptomatic heart failure [93].

### Myocardial fibrosis

It has been suggested that myocardial fibrosis may be the unifying pathophysiological process underlying CKD-associated cardiomyopathy [94]. In the 1990s, a post-mortem study found that myocardial fibrosis was present in 91% of CKD/KFRT patients without significant flow-limiting coronary lesions. The severity of fibrosis was related to the length of time on dialysis, but independent of hypertension, blood pressure, diabetes or anaemia [31]. However, given a plethora of drivers of cardiac fibrosis, as demonstrated by numerous preclinical and clinical studies, we cannot discount the contributing roles of these diseases on the activation of cardiac fibroblasts [95]. Over a decade later, Aoki et al. performed endocardial biopsies in 40 KFRT patients with reduced LV ejection fraction without coronary artery disease [15]. The predominant pathologic findings were extensive fibrosis and cardiomyocyte hypertrophy similar to that seen in the dilated phase of hypertrophic cardiomyopathy, a condition associated with extremely high morbidity and mortality [96, 97].

A post-mortem study of LV tissue from patients with KFRT found reduced capillary density, increased cardiomyocyte cross-sectional area and expansion of interstitial matrix. The apparent myocyte-capillary mismatch brought about by the reduction in capillary supply within a hypertrophied myocardium increases the oxygen diffusion distance, thereby exposing the heart to the risk of ischaemic injury and subsequent cardiac dysfunction [98]. Recent advances in imaging technology have enabled the indirect assessment of coronary microvascular dysfunction (CMD). Coronary flow reserve (CFR) is the most widely used surrogate marker, and has been successfully utilised to indirectly measure CMD in conditions such as hypertrophic cardiomyopathy and heart failure with preserved ejection fraction, both of which are characterised by LVH and myocardial fibrosis, similar to CKD-associated cardiomyopathy [99, 100]. Although data are limited, imaging studies utilising positron emission tomography, angiography and echocardiography have reported a prevalence of 24–90% in CKD patients with a graded relationship with eGFR [101]. In a retrospective study of CKD patients, which included those on dialysis, reduced CFR below 1.5 was associated with 2.1-fold increased risk of cardiovascular mortality independent of risk factors and LV function [102]. The mortality risk remained elevated in a later analysis of the dialysis-dependent cohort [103].

**Fig. 4:**
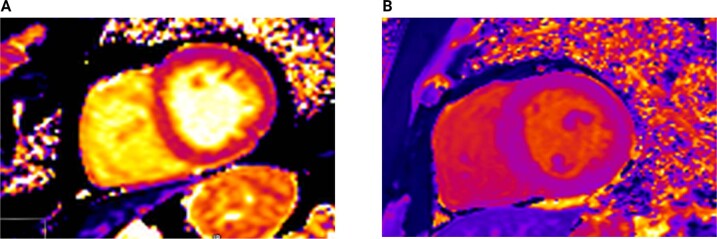
T1 cardiac magnetic resonance images demonstrating different levels of left ventricular fibrosis in dialysis patients. T1 mapping of the short axis stacked image of the mid left ventricular cavity (**A**) (Global T1 time 1385 ms) and (**B**) (Global T1 time 1170 ms) of 2 peritoneal dialysis patients consistent with high and low levels of cardiac fibrosis respectively.

Studying myocardial fibrosis in CKD/KFRT has been challenging given that myocardial biopsies are ethically difficult to justify [104, 105]. Contrast-enhanced cardiac magnetic resonance (CMR) imaging has been used for the assessment of myocardial fibrosis in conditions such as myocardial infarction [106], dilated [107] and hypertrophic cardiomyopathies [108]. Patients with KFRT demonstrate midwall patterns of late gadolinium enhancement consistent with replacement myocardial fibrosis not associated with large vessel coronary artery disease [36]. Non-contrast myocardial native T1 relaxation time, or T1 mapping, has emerged as a viable alternative for the assessment of myocardial fibrosis in CKD/KFRT [40] (Fig. 4). In cardiac tissues, T1 relaxation time outside regions of scarring has been shown to correlate with histologically measured interstitial fibrosis severity in a number of disease states [109]. In patients with KFRT, native T1 times are higher than age- and sex-matched controls, and correlate with increased LV mass [33, 34]. In parallel with the changes to LV mass and function, native T1 times have been shown to be increased in early CKD [32] and increase with worsening CKD stages [110] (Figs. 3 and 4). The contribution of myocardial water content to the increased T1 times remains contentious and to date, T1 times have not been validated with diffuse interstitial fibrosis in CKD-associated cardiomyopathy.

## Pathogenesis of CKD-associated cardiomyopathy

The pathogenesis of CKD-associated cardiomyopathy is likely to be multifactorial but can broadly be divided into three categories:

- Increased afterload [111],
- Increased preload [112],
- Intrinsic factors not directly to afterload or preload [113].

**Fig. 5:**
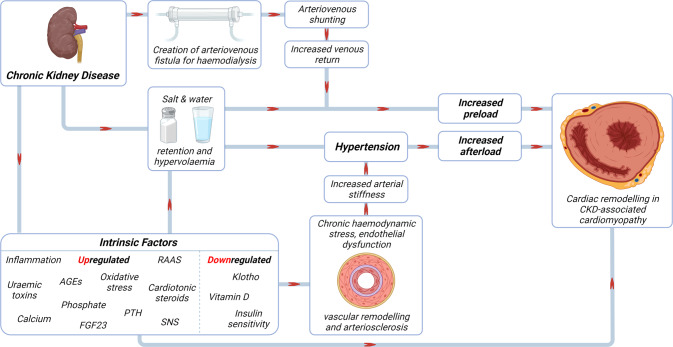
Pathogenesis of CKD-associated cardiomyopathy. An interplay of pathophysiological processes associated with the chronic kidney disease milieu contribute to the pathogenesis of CKD-associated cardiomyopathy. These can be broadly divided into factors which increase afterload, preload, and those which result from maladaptive perturbations of intrinsic factors. AGEs advanced glycation end-products, FGF23 fibroblast growth factor 23, PTH parathyroid hormone, RAAS renin-angiotensin-aldosterone system, SNS sympathetic nervous system.

Although these categories are considered separately, it is important to note that there is considerable overlap between them [40, 41, 101, 114, 115]. Factors within these categories may indeed interact and exert their effects synergistically (Fig. 5).

### Increased afterload

Raised intra-arterial pressure induces cardiac hypertrophy through chronic haemodynamic stress on the myocardium. In vitro experimental models of cardiomyocyte and non-myocyte stretch resulted in cell hypertrophy and hyperplasia, induction of protein synthesis and increased expression of prohypertrophic proto-oncogenes such as fos, jun, myc, Ha-ras, and genes encoding atrial natriuretic factor and β-myosin heavy chain [116–118]. This was recapitulated in murine and porcine studies of hypertension and volume overload where transcription of prohypertrophic genes, and development of LVH were observed [119–123].

#### Arterial stiffness/decreased large vessel compliance/vascular calcification

Arterial stiffening occurs from the early stages of CKD and increases with the progression to KFRT [124–126]. The extent of arterial stiffness can be reliably estimated by non-invasive measurement of carotid-femoral pulse wave velocity (PWV), which can confer prognostic value in CKD patients independent of traditional risk factors [127, 128].

Arterial stiffness is both a cause and result of systolic hypertension, although extricating the bidirectional relationship can be challenging [129, 130]. Studies have demonstrated increased arterial stiffness as a consequence of increased distending vascular wall stress and remodelling from hypertension itself, so called pressure-dependent increases in PWV [131–133]. Other studies show that increased arterial stiffness, more specifically higher PWV, predicts future development of hypertension [134–137], possibly as a result of inherent pressure-independent vasculopathy. In patients with KFRT, PWV is independently associated with nonfatal stroke and myocardial infarction [126] and with cardiovascular and all-cause mortality [126, 138, 139]. Of those patients who died, it was found that the persistence of increased PWV despite a reduction in blood pressure by haemodialytic and pharmacological strategies (angiotensin converting enzyme inhibitor; ACEi) was a significant independent risk factor. This is in contrast to the surviving patients where a similar fall in blood pressure was mirrored by a reduction in PWV [139].

In addition to elevated blood pressure, there are several other postulated pathophysiological drivers of increased arterial stiffness in CKD such as activation of the renin-angiotensin-aldosterone system (RAAS), build-up of uraemic toxins, disordered bone and mineral metabolism (CKD-MBD), accumulation of advanced glycation end-products, chronic inflammation and oxidative stress which adversely affect endothelial function and calcific remodelling of the vessel wall [12, 129]. The reduced vessel compliance is manifested by hypertension and wide pulse pressure, and disruptions to end-organ perfusion, resulting in myocardial fibrosis, increased LV mass/LVH and diastolic dysfunction bearing the hallmarks of CKD-associated cardiomyopathy [140]. Taken together, blood pressure reduction alone without ‘de-stiffening’ of the arteries may be insufficient to reduce risk of death in patients with CKD/KRFT.

### Increased preload

Intravascular volume expansion/hypervolaemia, secondary to salt and water loading, is a frequent occurrence in CKD/KRFT and a determinant of LV mass and mortality [141, 142]. Indeed, volume expansion is a major contributor to the hypertension observed in patients with CKD/KRFT [142, 143]. Reduced GFR, activation of the RAAS as well as superimposed cardiovascular disease all contribute to sodium and water retention in these patients [144–146].

As haemoglobin concentrations fall, complex haemodynamic compensatory mechanisms are activated to increase cardiac output and blood flow to compensate for tissue hypoxia [147]. These include a reduction in afterload secondary to reduced systemic vascular resistance, an increase in preload secondary to increased venous return, and increased LV function as a consequence of increased sympathetic tone [148, 149]. Treatment of anaemia in CKD with erythropoietin stimulating agents (ESAs) reduces stroke volume and cardiac output as a consequence of both reduced venous return and increased peripheral resistance [150–153]. These functional changes are paralleled by structural changes in the LV, with partial reduction of LVH [151, 152]. It should also be noted that treatment with ESA increases blood pressure by direct vasomotor effects [154–156].

The use of arteriovenous fistulas for haemodialysis is preferrable to vascular catheters due to improved dialysis quality and reduced infection rates [157–159]. However, the creation of a shunt between a high pressure arterial vasculature and a low resistance venous system increases cardiac output through the shunting itself, activation of the sympathetic nervous system, neurohormonal changes, and increased venous return [158]. Chronically increased preload and cardiac output lead to LVH and right ventricular remodelling, which in turn is associated with an 3.9-fold increased risk of death [160]. Several studies of fistulas being tied off after successful kidney transplantation show some evidence of LVH regression [161, 162]. However, although creation of an arteriovenous fistula decreases systemic blood pressure, ligation increases systolic blood pressure by an average of 5 mmHg [163]. The decision to proceed with fistula ligation should therefore balance, on an individualised basis, the benefits of cardiac remodelling regression against the potential adverse effect on blood pressure control.

### Intrinsic factors

In addition to the haemodynamic factors already discussed, several other factors that become dysregulated in patients with CKD are involved in the pathogenesis of CKD-associated cardiomyopathy. These include activation of RAAS, sympathetic nervous system overactivity, increased transforming growth factor-beta (TGFß) signalling, insulin resistance, uraemic toxins (e.g., indoxyl sulfate, p-cresyl sulfate), increase in cardiotonic steroids, oxidative stress and factors associated with CKD-MBD namely hyperparathyroidism, vitamin D deficiency, increased circulating fibroblast growth factor-23 (FGF-23), decreased Klotho expression and hyperphosphataemia [41, 164–166]. Although often considered in isolation, there are significant overlaps and crosstalk between these pathways. For example, FGF-23 activates RAAS through suppression of ACE2 [167], which downregulates vasodilatory angiotensin- [1–7], and upregulates plasma/cardiac Angiotensin II and aldosterone [168–170]. In turn, aldosterone stimulates FGF-23 transcription in osteoblasts in vitro, an effect reversed by mineralocorticoid receptor blockade [171]. More recently, Böckmann et al. showed that FGF23 stimulated expression of RAAS genes and hypertrophic growth in cultured neonatal rat ventricular myocytes [172].

## Treatment of CKD-associated cardiomyopathy

Given that CKD-associated cardiomyopathy, and its individual components, are powerful predictors of cardiovascular mortality in patients with CKD and KFRT, targeting the mechanisms involved seems a practical approach to improve outcomes. However, a meta-analysis assessing the validity of LV mass reduction as a surrogate marker of all-cause and cardiovascular-mortality in patients with CKD and KFRT concluded there was no clear association between intervention-induced change in LV mass and mortality [173]. It should be noted that, by the authors’ own admission, most of the included trials were of short duration with small sample sizes, with either an uncertain or high risk of bias, and sparse mortality data [173].

### Dialysis treatments

**Fig. 6:**
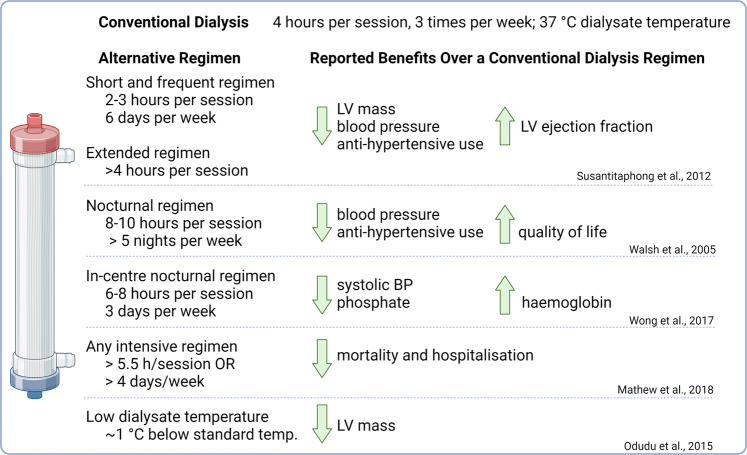
Effect of dialysis regimen on blood pressure, cardiac and other outcomes. Conventional haemodialysis regimens consist of a four-hour haemodialysis session delivered on alternate days, three times per week. Longer, more frequent dialysis regimens have been trialled, with benefits on blood pressure, left ventricular mass, mortality, hospitalisations, and quality of life. However, the implementation of such ‘intensive’ regimens significantly increases the demand on healthcare providers and may be associated with risks of infection, vascular access complications and loss of residual kidney function.

Studies have shown that longer, frequent ‘intensive’ dialysis regimens (Fig. 6) are associated with reductions in LV mass, lower prevalence of LVH and reduced hospitalisations [174–177]. However, it is not clear if the reductions in LV mass are a direct consequence of the dialysis treatment - clearance of uraemic toxins, better phosphate and haemoglobin control; or improved blood pressure control [178–180]. An intriguing, but small, study of 54 incident haemodialysis patients found that lowering dialysate temperature by an average of 1 °C significantly reduced LV mass (treatment difference between groups 15.6, 95% CI 1.9–29.4 g) measured by CMR over a 12 month period with no differences in blood pressure between groups [181]. However, 10 of the 54 patients had their 12-month CMR results imputed. This study finding requires confirmation in larger trials.

The previously discussed effects of intensive dialysis on reduction of hypertension and improvement in LV mass are encouraging, however it is still unclear as to whether this leads to better patient survival. A recent meta-analysis encompassing 70,506 patients found that intensive dialysis was associated with reduced mortality when compared to conventional dialysis, but the overall quality of the evidence was low [182]. Whilst intensive dialysis has clear health and quality of life benefits, it needs to be considered in conjunction with the potential risks of increased patient and caregiver burden, infection, loss of residual kidney function and vascular access complications [183].

### Transplantation

The gold-standard for the treatment of KFRT is kidney transplantation [184]. The associated improvement in GFR reduces cardiovascular risk below that of those on waiting lists [185]. However, cardiovascular risk still remains higher than healthy individuals of the same age and sex with transplant recipients displaying a three-fold increased risk [186]. The restoration of kidney function associated with kidney transplantation improves many factors thought to cause CKD-associated cardiomyopathy. As a result it is generally assumed that kidney transplantation reduces LV mass, and improves diastolic and systolic function, and potentially reverses myocardial fibrosis [187, 188]. Indeed, review articles continue to state that CKD-associated cardiomyopathy is reversed by kidney transplantation. However, these articles frequently will either not cite any references [41], cite small, uncontrolled studies using either echocardiography [189] or radionucleotide ventriculography-gated blood pool scans [187, 190], or refer to other review articles [191].

A recent systematic review and meta-analysis has indeed confirmed that many echocardiographic studies reported significant reductions in LV mass after kidney transplantation [192]. However, this study highlighted key problems in the available literature. Few studies were blinded or had a control group thus were prone to bias; meta-analysis of the four studies containing a control group did not find any association between transplantation and LV mass. Echocardiography is unreliable for LV mass determination in dialysis patients [193]; few studies used CMR which is more reproducible in KFRT patients [40]. Indeed, none of the three CMR studies included in the review found a significant change in LV mass.

Intriguingly, a small study in 44 kidney transplant recipients found a significant reduction in native T1 time at 6 months after transplantation with no reduction in LV mass [194] A smaller study found no difference in global T1 times 6 weeks after transplantation suggesting changes may continue to progress over time [195]. Larger, controlled studies utilising CMR are required to further investigate this fundamental question.

### Blood pressure treatment

#### Blood pressure target in chronic kidney disease

Prior to publication of the SPRINT trial results [196], most societies and guidelines recommend lowering blood pressure to below 140/90 mmHg [197–201] with some suggesting higher thresholds for the elderly [197, 198, 201] and lower thresholds for those at higher high risk including patients with diabetic mellitus and patients with CKD [197, 198, 201]. It is now recognised that more intensive blood pressure reduction strategies are effective and safe. The SPRINT investigators reported a 25% lower risk of major cardiovascular events such as myocardial infarction, heart failure and stroke and 27% lower risk of all-cause mortality when treating to a target systolic blood pressure of less than 120 mmHg compared to the standard target of 140 mmHg. This was associated with an increase in complications such as lower serum potassium, sodium, and syncopal episodes, but no increased risk of falls. Treatment of hypertension in those with CKD, which formed 28% of the SPRINT cohort, was associated with improved cardiovascular outcomes and all-cause mortality, with no adverse effects on kidney function or increased risk of dialysis. Elderly patients aged over 75 years old also sustained benefit from more intensive blood pressure control [196].

**Fig. 7:**
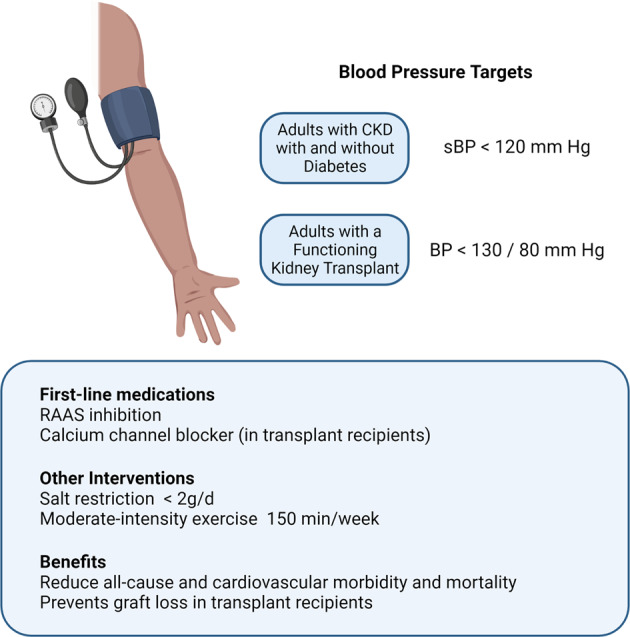
Blood pressure management in chronic kidney disease. The recently updated Kidney Diseases Improving Global Outcomes (KDIGO) 2021 Blood Pressure Guideline provides clear recommendations for the management and benefits of blood pressure control in patients with CKD (incl. transplant recipients).

These data have resulted in lowering of both American and European blood pressure targets in their respective 2017 [202] and 2018 [203] hypertension guidelines. The recently published Kidney Disease Improving Global Outcomes (KDIGO) 2021 guideline on the management of blood pressure in CKD also recommends an intensive systolic blood pressure target of 120 mmHg or less in patients with CKD (excluding those with a functioning kidney transplant) with an emphasis on standardised office blood pressure measurement and lifestyle interventions such as salt restriction and moderate-intensity exercise [204] (Fig. 7).

#### Efficacy of blood pressure treatment on left ventricular hypertrophy

**Fig. 8:**
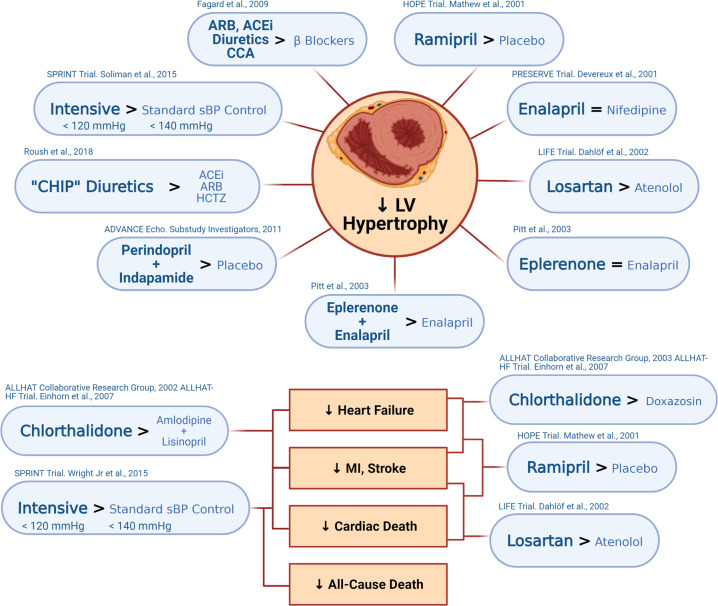
Comparing the cardiac effects of conventional antihypertensive therapy. Several landmark trials and meta-analyses have compared the efficacy of antihypertensive drug classes on cardiac functional, structural, and mortality endpoints.

In the general population, regression of LVH is associated with a significant improvement in cardiovascular events [205]. Several landmark trials have confirmed that LV regression in response to treatment with antihypertensive medication is associated with significant reduction in cardiovascular events [206–209]. All classes of anti-hypertensive medications have been shown to regress LVH [210–213]. In general, meta-analyses have shown that ACE-inhibitors, angiotensin receptor blockers (ARBs) and calcium channel blockers are more effective than beta-blockers at regressing LVH [214, 215]. Multiple studies have shown that “CHIP” diuretics (chlorthalidone, indapamide and potassium-sparing diuretics/hydrochlorothiazide) are more effective than ACE-inhibitors/ARBs, as well as hydrochlorothiazide, at reducing LV mass [216–218] (Fig. 8).

Factors associated with persistent LVH and lack of regression of LVH with antihypertensive treatment include older age, higher body mass index, sub-optimal blood pressure control, duration of hypertension and crucially, the presence of CKD [210, 219, 220]. This, together with the fewer and much smaller studies available make the literature in patients with CKD/KRFT less clear. Most, but not all [221, 222] studies of ACEi/ARB compared with placebo or standard treatment in both patients with CKD [223] or on dialysis [224–227] have shown no significant reduction in LVM. The same is true for studies comparing ACEi/ARB with a CCB [228–230] or beta-blocker [231, 232] which have shown no difference between agents.

The confounding effect of blood pressure reduction on changes in LV mass are highlighted by a study examining the cardiovascular effects of spironolactone compared with placebo in early-stage CKD [233]. In 112 non-diabetic patients with CKD stages 2–3 and well-controlled blood pressure, the addition of spironolactone 25 mg once daily for 40 weeks reduced LV mass by 14 g compared with a non-significant change of 3 g with placebo. However, significantly greater falls in blood pressure were observed in the spironolactone group. A subsequent study in 154 patients, with similar characteristics to the original trial, found no significant difference in the reduction of LV mass observed with spironolactone compared with chlortalidone over a 40-week period [234]. Importantly, reductions in office and 24-hour blood pressure readings were not different between groups either.

#### Angiotensin receptor neprilysin inhibitor

Sacubitril/valsartan are the first in this new class of angiotensin receptor neprilysin inhibitors (ARNI), approved for the treatment of heart failure with reduced ejection fraction (HFrEF) in conjunction with other standard therapies [235]. The neprilysin inhibitor sacubitril enhances the activity of the natriuretic peptides which have a counter-regulatory role in conditions linked to RAAS activation such as heart failure and CKD through increased sodium & water excretion [236, 237]. Its use in combination with valsartan prevents reflex activation of RAAS [238]. Clinical studies have shown sacubitril/valsartan to reduce the risk of death and hospitalisation when compared to enalapril [239], and provide a further reduction to blood pressure versus valsartan alone [240]. In patients with CKD and HFrEF, a meta-analysis of randomised control trials comparing 3,460 patients on an ARNI versus a RAAS-blocker found that ARNIs significantly reduced blood pressure and N-terminal pro-brain natriuretic peptide (NT-proBNP, and was mildly renoprotective [241]. Patients with CKD can present with diastolic dysfunction only, or heart failure with preserved ejection fraction (HFpEF). The efficacy of ARNIs in HFpEF is less convincing. PARAGON-HF studied 4,796 patients with LV ejection fraction ≥45% and reported no significant change in heart failure hospitalisations and cardiovascular death when compared to valsartan alone [242]. In the PARALLAX trial, ARNI did not increase functional- or symptom-based scores despite a 16% reduction in NT-proBNP versus RAAS inhibition/placebo [243].

#### Mineralocorticoid receptor antagonists

It is well-established that aldosterone promotes cardiovascular damage and high concentrations are associated with LVH [244, 245] and myocardial fibrosis both in experimental and human studies [246, 247]. The mineralocorticoid receptor (MR) is expressed in vascular smooth muscle, as well as cardiomyocytes and myofibroblasts [248], and may directly stimulate proliferation of myofibroblasts [249]. Aldosterone directly induces cardiac hypertrophy, ventricular remodelling, arrhythmia and ischaemia, independently of its hemodynamic effects [250] and it appears that progression from LVH to cardiac failure is mediated by aldosterone through the MR [251]. Mineralocorticoid receptor antagonists (MRA) have consistently shown beneficial effects on left ventricular dilation, cardiac function, fibrosis or collagen content in preclinical studies [252–254] and also regress LVH in clinical trials [212]. MRA have consistently been shown to improve outcomes in patients with HFrEF and potentially in patients with HFpEF [255].

Whether the lowering of LV mass in CKD patients is dependent on the blood pressure lowering effect of MRAs is unclear [233, 234]. Recently two large placebo-controlled studies with the non-steroidal MRA finerenone have shown significant reductions in cardiovascular events and mortality in patients with diabetic nephropathy with little effect on blood pressure [256–258]. Consistent with the effect of blood pressure on stroke risk [203, 259] the incidence of stroke did not vary between groups [255]. The lower incidence of hyperkalaemia versus steroidal MRAs (spironolactone and eplerenone) makes finerenone an attractive option for the treatment of CKD-associated cardiomyopathy [255].

#### Sodium-glucose co-transporter-2 inhibitors

**Fig. 9:**
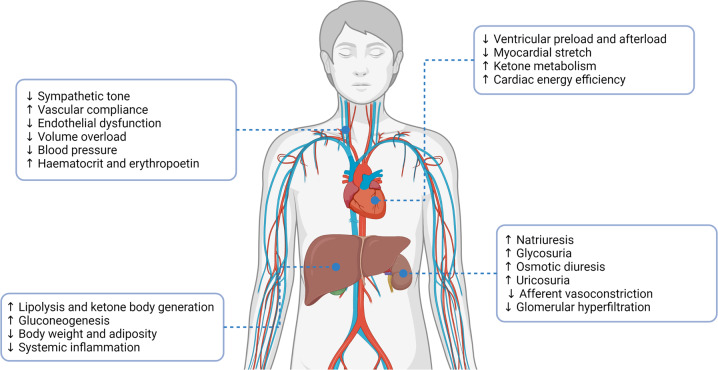
Pleiotropic effects of sodium-glucose cotransporter type 2 inhibition. Sodium-glucose cotransporter type 2 inhibitors have traditionally been an effective antidiabetic therapy. Recent trials have demonstrated a multitude of beneficial cardiorenal, metabolic and vascular effects, which underpin the improvement in cardiovascular and renal outcomes independent of improved glycaemic control. Some of these mechanisms have been summarised.

Sodium-glucose co-transporter-2 (SGLT2) inhibitors are a recent class of oral antidiabetic agents [260, 261]. They inhibit renal glucose reabsorption and several SGLT2-inhibitors are licensed in various countries for the treatment of diabetes mellitus. In addition SGLT2-inhibitors significantly reduce weight through glycosuria-associated calorie loss [260, 261]. They also decrease sodium-reabsorption exhibiting a mild natriuretic and diuretic effect [260, 261] (Fig. 9).

A recent meta-analysis of large RCTs clearly demonstrated a 23% reduction in CV morbidity and mortality, especially heart failure hospitalisations in patients with diabetes mellitus [262]. Similar results are emerging in patients with CKD and with heart failure without diabetes [263, 264]. Short-term, mechanistic studies have shown that SGLT2-inhibitors reduce LV mass in diabetics with [265] and without LVH [266]. These early effects of SGLT2-inhibitors on LV remodelling are consistent with their rapid impact on cardiovascular death and heart failure hospitalisation observed in the trials [267]. In addition SGLT2-inhibitors have been shown to improve LV diastolic dysfunction [268, 269].

Several mechanisms have been proposed for the reduction in LV mass and improved diastolic dysfunction observed with SGLT2-inhibitors [267] (Fig. 9). Their effects on lowering intracellular sodium concentrations in the heart have however recently been disproved [270, 271]. It should be noted that SGLT2-inhibitors also significantly lower systolic and diastolic blood pressure by 3–5/1–2 mmHg [261], through their diuretic and natriuretic actions, as well as reported reductions in sympathetic activity, arterial stiffness and vascular resistance [260, 261] (Fig. 9). A significant correlation between blood pressure reduction and regression of LVH with SGLT2-inhibitor use has been reported [269]. SGLT2-inhibitors might therefore be useful agents to improve CKD-associated cardiomyopathy acting via both blood pressure reduction and blood pressure independent mechanisms.

#### Renal sympathetic denervation

Hypertension can be challenging to control in CKD, with the prevalence of apparent resistant hypertension reported to be as high as 40% [272]. Whilst the true prevalence is likely to be lower after accounting for medicines nonadherence and white coat syndrome, patients with true resistant hypertension are at increased risk of adverse cardiovascular outcomes compared with those who are treatment-responsive [273]. Following initial disappointment [274], several recent randomised sham-controlled trials have now shown that renal denervation therapy can effectively reduce blood pressure [275–279]. The latest analyses of 3-year data from the Global SYMPLICITY Registry provide encouraging news for patients with CKD, who experience a similar reduction in blood pressure following renal denervation to those without renal disease, and also demonstrate the safety of the procedure [280, 281]. The longer-term effects on renal function and cardiovascular events are yet to be determined, with trials currently ongoing (NCT01888315, NCT04264403).

### Treating individual factors

#### Anaemia

The presence of anaemia is associated with the development of LVHH and heart failure in patients with CKD [282, 283], KFRT [284, 285], and kidney transplant recipients [286, 287]. Correction of severe anaemia with ESA is associated with a reduction in LV mass [288]. However, correction of moderate anaemia to target haemoglobins above 12 g/dL appears to have no effect on LV mass [288]. Interestingly, the correction/normalisation of anaemia to targets above 12 g/dL have consistently been shown to increase the risk of myocardial infarction, heart failure and all-cause mortality in patients with CKD and KFRT [289–292].

#### Factors associated with CKD-MBD

**Fig. 10:**
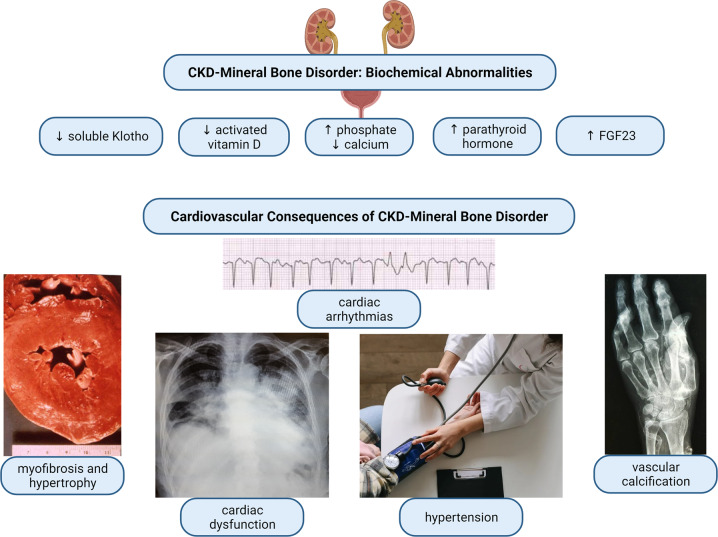
Cardiovascular consequences of CKD-MBD. Chronic kidney disease – mineral bone disorder (CKD-MBD) describes the alterations in circulating and tissue levels of calcium and phosphate because of changes in parathyroid hormone, vitamin D, fibroblast growth factor 23 (FGF23) and its coreceptor Klotho. These biochemical changes are associated with the cardiovascular remodelling and clinical manifestations observed in patients with CKD.

CKD-MBD encompasses the progressive deterioration in the homoeostasis of calcium, phosphate due to disruption in circulating hormones FGF23 and its co-receptor Klotho, PTH and vitamin D (Fig. 10). Higher circulating levels of phosphate are associated with higher LV mass and LVH in the general population, patients with CKD and in dialysis patients [164, 293, 294]. However, the relationship between serum phosphate and increased LV mass has not yet been proven to be causal although a small, recent study reported a significant association between reduction in serum phosphate and regression of LV mass [295].

Low levels of vitamin D have been associated with higher blood pressure [296], a higher incidence of hypertension [297], and LVH, possibly mediated by parathyroid hormone [298, 299]. Patients with CKD often develop vitamin D (1,25-dihydroxyvitamin D_3_/calcitriol) deficiency because of a lack of its precursor, 25-hydroxyvitamin D3 and impaired activity of the kidney enzyme 1α-hydroxylase [300]. Observational studies have suggested a beneficial association between therapy with calcitriol or related analogues and reduced cardiovascular events [301–305] with experimental models suggesting that these actions are mediated by a reduction in LVH, and improved LV diastolic function [299, 306–309]. However, to date, randomised controlled trials have not shown a significant reduction in either blood pressure or LVH with vitamin D treatment [310, 311].

The phosphaturic hormone FGF23 is markedly increased in patients with CKD, and dialysis [165, 312–314] and has been causally linked to the development of LVH [315] and LV dysfunction [316] and arrythmias such as AF [317]. The calcimimetic agent cinacalcet reduces circulating FGF23 and has been shown to reduce cardiovascular death, sudden cardiac death and heart failure in patients on dialysis [318]. However, it should be noted that calcimimetics appear to have a consistent blood pressure lowering effect in experimental models of uraemia and patients with CKD and KFRT [295, 319–322].

The FGF23 co-receptor αKlotho is a protein severely downregulated in CKD [323] due to vitamin D deficiency [324, 325], RAAS overactivity [326, 327], inflammation [328] and also by FGF23 excess [329]. In haemodialysis patients, αKlotho deficiency is a strong predictor of cardiovascular events and mortality [330, 331]. In its secreted, circulating form, soluble αKlotho exerts a broad array of biological functions as evidenced in studies of αKlotho-deficient mice which manifested an ageing phenotype of phosphate imbalance, osteoporosis/osteopenia [332], vascular calcification, growth retardation, emphysema [333] and premature death [334], cardiac hypertrophy and fibrosis [335–337]. In addition, αKlotho has a role in blood pressure homoeostasis. In a study of 2774 subjects, higher αKlotho was associated with a lower risk of incident hypertension [338]. Preclinical studies have shed light on the potential underlying mechanisms, including αKlotho inhibition of inflammation-associated renal sodium retention [339, 340] and endothelial dysfunction through reduced oxidative stress and increased nitric oxide availability [341–343] in murine and in vitro studies. Klotho supplementation in uraemic and diabetic mice protected against uraemic toxin [344], angiotensin II [345], inflammation [345, 346], oxidative stress [346] and myocardial FGF21-induced LVH [347]. The use of recombinant αKlotho in the treatment of hypertension and CKD-associated cardiomyopathy remains a promising therapeutic option [348] although yet to trialled in larger mammals.

#### Weight loss

Obesity, defined as a body mass index of ≥30 kg/m^2^, is a strong predictor of metabolic syndrome and plays a critical role in the development of cardiovascular and kidney disease [349–353]. In a meta-analysis of 1022 obese patients with preserved systolic function, surgical bariatric interventions resulted in a decrease in LV mass and improved LV diastolic function during a mean follow-up period of 16 months [354]. Whilst lifestyle interventions can be effective in the short term, bariatric procedures have been proven to result in more persistent weight loss compared to non-surgical approaches [355]. A randomised controlled trial of 41 hypertensive, obese patients found that a mean weight loss of 8.3 kg achieved through lifestyle changes led to a 16% reduction (adjusted for body surface area) in LV mass, independent of blood pressure changes [356]. This is particularly intriguing as weight loss has also been associated with reduction in proteinuria and improved renal function [357–359]. However, data on the longer-term outcomes of lifestyle interventions in obese, diabetic individuals demonstrate no significant difference in cardiovascular events at 10 [360] and 20 [361] years of follow-up. Although few in number, recent observational studies reported significantly lower rates of cardiovascular events and death in obese patients 5–15 years following bariatric surgery [362–364]. How these data can be extrapolated to patients with kidney disease remains to be investigated.

## Conclusions and future perspectives

The pathophysiology of CKD-associated cardiomyopathy is extremely complex and involves multiple inter-related mechanisms. Increasing evidence linking individual factors to its aetiology may well lead to novel treatments in the future. However, the current evidence continues to suggest that the diagnosis, characterisation, and optimisation of treatment of hypertension is the cornerstone of treatment for now. To date, specific, blood pressure independent, beneficial treatment effects on either LV mass or hard clinical end points have not been convincingly demonstrated. While further research into potential treatments targeting molecular aetiological pathways needs to continue, research into the optimal blood pressure targets, monitoring strategies and antihypertensive regimens, for individuals at different stages of CKD and with KFRT needs to progress in parallel.
